# Spontaneous intramuscular hemorrhage in cancer-associated dermatomyositis: a case and literature review

**DOI:** 10.1186/s12891-023-06651-z

**Published:** 2023-07-01

**Authors:** Rui Xing, Fenfen Xiang, Lingli Dong, Guifen Shen

**Affiliations:** 1 Department of Rheumatology and Immunology, Tongji Hospital, Tongji Medical College, Huazhong University of Science and Technology, Jiefang Avenue 1095, Wuhan, 430030 Hubei Province China; 2 Department of Endocrinology, Rheumatology and Immunology, Huanggang Central Hospital of Yangtze University, Huanggang, 438000 Hubei Province China

**Keywords:** Dermatomyositis, Spontaneous intramuscular hemorrhage, Hematoma, TAT, PIC, t-PAIC, Signet-ring cell carcinoma

## Abstract

**Background:**

Spontaneous intramuscular hemorrhage (SIH) is a rare but life-threatening complication of dermatomyositis (DM). The pathogenetic mechanism and management of intramuscular hematoma in these patients remains unclear. Here we discuss a case of recurrent hemorrhage in a patient with cancer-associated DM, and review the relevant literature for timely diagnosis and treatment.

**Case presentation:**

A 53-year-old male patient presented with rashes, muscle weakness, and dysphagia and was diagnosed with DM. During treatment, he developed SIH of the arm and right psoas major muscle successively. MRI showed extensive edema of the right shoulder girdle muscle and muscle groups of the upper arm. During the second SIH, a CT scan showed new-onset hematoma formation in the right psoas major muscle. The detection of D-dimer, thrombin-antithrombin III complex (TAT), plasmin-α2-plasmininhibitor complex (PIC) and tissue plasminogen activator-inhibitor complex (t-PAIC) indicated predominant hyperfibrinolysis over thrombosis. Blood transfusion and supportive treatment were immediately performed, and the hematoma did not expand. However, his abdominal distension was not relieved after active treatment. Further electronic gastroscopy discovered gastric sinus ulcers, and histopathology of the biopsy confirmed signet-ring cell carcinoma.

**Conclusions:**

Although patients with cancer-associated DM have an increased risk of thrombosis, prophylactic anticoagulation therapy needs deliberate consideration. It is important to monitor the coagulation parameters dynamically during anticoagulation therapy. Especially when the level of D-dimer is high, and it is uncertain whether the patient is in a state of thrombosis or hyperfibrinolysis, the detection of TAT, PIC, t-PAIC can help to determine whether to initiate anticoagulation therapy.

**Supplementary Information:**

The online version contains supplementary material available at 10.1186/s12891-023-06651-z.

## Background

Dermatomyositis (DM), one kind of idiopathic inflammatory myopathy (IIM), is a systemic autoimmune disease with characteristic cutaneous manifestations and muscle weakness of the trunk and symmetrical proximal muscles of the limbs. Characteristic rashes include Gottron’s papules and heliotrope erythema. In addition to muscle and skin involvement, other organs can also be involved, of which interstitial lung disease (ILD) and constitutional symptoms such as fever are also common in DM [1]. IIMs are associated with an increased incidence of malignancy, and DM has a higher incidence of combined malignancy than polymyositis. A meta-analysis of 5 studies with 4538 patients showed that the relative risk of cancer was increased 4.66-fold in patients with DM compared to the normal population [2]. Previous studies have shown that the most common cancers in patients with DM are lymphatic and hematopoietic system cancer, followed by lung cancer [3].

DM is a multisystem-affected disease that can lead to many serious complications, such as rapidly progressive ILD, macrophage activation syndrome (MAS), pneumomediastinum, and spontaneous intramuscular hemorrhage (SIH) [4, 5]. SIH, also called hemorrhagic myositis, is a rare but life-threatening complication of DM. It presents as short-term, insidious, and nontraumatic muscular hemorrhage or hematoma, which may cause disseminated intravascular coagulation (DIC) or hemorrhagic shock [5]. Until now, the pathogenetic mechanism and management of intramuscular hematoma in these patients have remained unclear. Here, we report a case of recurrent hemorrhage in a patient with cancer-associated DM and review the relevant literature for timely diagnosis and treatment.

## Case presentation

A 53-year-old male patient complained of weakness and myalgia with rashes for a half month. His skin rashes had spread from the face, scalp, chest, and back to hips, with itching, and the symptoms increased after exposure to sunshine. His generalized fatigue and weakness worsened, which was accompanied by dyspnea, dysmasesis, and dysphagia. Physical examination showed that the muscle strength of the neck flexors and proximal upper and lower limbs was 3/5. Erythematous patches over the scalp, minimal heliotrope rash, and fuscous rashes over his chest, back and hips were discovered during the skin exam. He had a history of perianal abscess surgery 5 years prior, gastrointestinal polyp resection 3 months prior, and left hip replacement due to avascular necrosis of the femoral head one month prior.

**Fig. 1:**
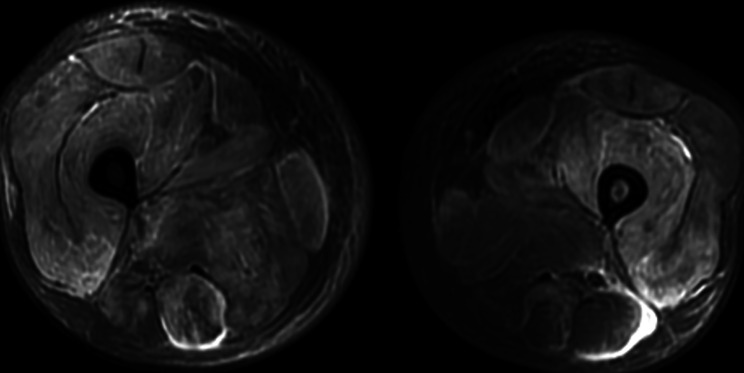
Representative magnetic resonance imaging (MRI) images of diffuse edema in the thigh muscle. Bilateral edema of all thigh muscles and intermuscular fascia and subcutaneous soft tissue edema

Laboratory tests showed a minor positive ANA 1:100, and myositis-specific antibodies (MSAs) and myositis-associated antibodies (MAAs), extractable nuclear antigen (ENA), and anti-neutrophil cytoplasmic antibodies (ANCAs) were all negative. Other laboratory findings included extremely elevated creatine kinase 8875 U/L (ref ≤ 190), with increased ALT 125 U/L, AST 413 U/L, LDH 940 U/L, ferritin 2573.8 ug/L (ref 30–400), CEA 10.70 ng/ml (ref ≤ 5.0), IL-6 8.41 pg/ml (ref 0.1–2.9) and decreased C3 0.73 g/L (ref 0.8–1.8). His blood hemoglobin (HB) (136 g/L) and platelet count were normal. Basic coagulation testing revealed elevated D Dimer 5.91 µg/ml FEU (ref < 0.5) with normal PT, APTT, and fibrinogen. Magnetic resonance imaging (MRI) detected diffuse edema in the bilateral thigh muscle groups and intermuscular fascia (Fig. 1). Electroneuromyography (EMG) demonstrated electrophysiological features of multiple types of myogenic damage. Positron emission tomography-computed tomography (PET-CT) was performed given that the patient had unusually high levels of CA19-9 (400.60 U/ml) and CEA (10.7 ng/ml). PET-CT revealed that muscle metabolism was increased (both upper extremities, the right pectoral muscles, and bilateral scapular muscles were the most prominent and swollen); and the retroperitoneal lymph nodes were increased in size and metabolism. Based on these findings, he was diagnosed with DM.

**Fig. 2:**
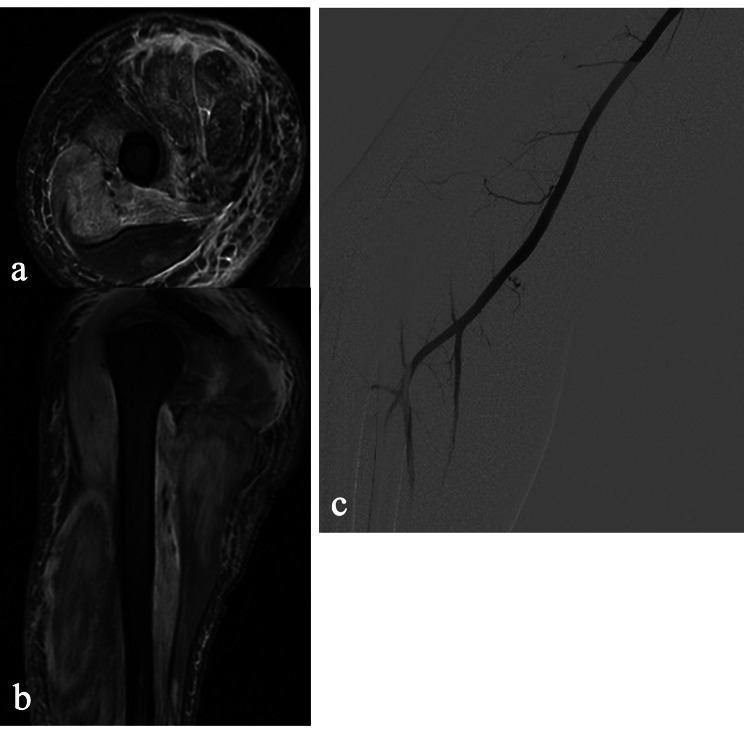
Representative images of the intramuscular hematomas in the right arm MRI images of the extensive edema of the right shoulder girdle muscle and muscle groups of the upper arm (**a, b**). Angiography shows a small branch of the brachial artery was bleeding (**c**)

**Fig. 3:**
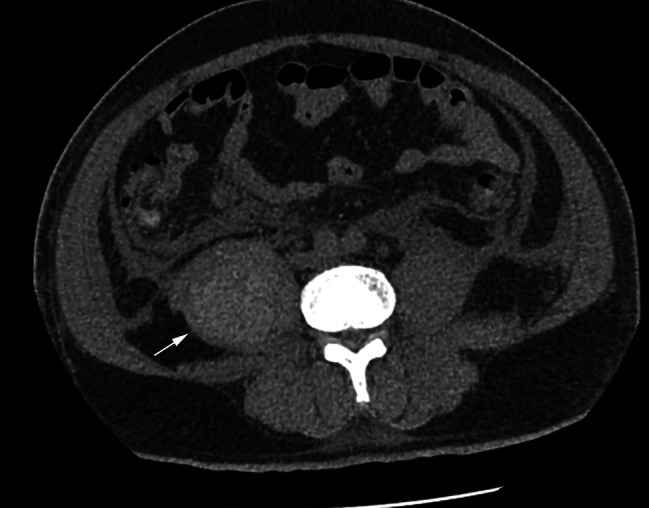
Representative computed tomography (CT) images of the intramuscular hematomas in the right psoas major muscle The CT image showed a lamellar slightly dense shadow of the right psoas major muscle mass (arrow)

On admission, he was started on pulse methylprednisolone 200 mg per day for 5 days, since he was treated with prednisone 40 mg per day without improvement before admission, plasmapheresis for one time, and prophylactic low molecular weight heparin (LMWH) 3200 IU per day. On Day 3, he presented with severe pain in the right arm. Swelling of the right arm and hand was found with purple bruises. He also had pain in both shoulders and right chest. Ultrasound showed that the muscle fibres in the right upper extremity were swollen, and liquid dark areas were seen locally (consider hematoma). MRI showed extensive edema of the right shoulder girdle muscle and muscle groups of the upper arm (Fig. 2a and b). APTT was slightly prolonged (50.2 s), with decreased fibrinogen (1.33 g/L), and HB had dropped from 136 g/L to 82 g/L, platelet decreased from 125 × 10^9^/L to 67 × 10^9^/L (Table 1). Emergent angiography confirmed that a small branch of the brachial artery was bleeding (Fig. 2c). Since embolism therapy and surgery were impropriate in that situation, a conservative pressure dressing treatment was placed (Additional Figure S1). LMWH was stopped and transfusion with red blood cells and fresh frozen plasma was supplied with hemostatic therapy. The bleeding was controlled after therapy. He remained stable for 10 days. Due to critical dysphagia and weakness, pulse IVIG 20 g per day for 5 days was given. Considering that the increased D-dimer level (10.76 µg/ml) and prolonged recumbency increased the risk of venous thromboembolism (VTE), prophylactic LMWH was started again. The weakness was not worse, while the creatine kinase (CK) level decreased to 1831 U/L. However, 4 days later, he complained of abdominal distention and gradual right thigh pain. MRI showed hematoma formation in the right psoas major muscle, and the CT scan showed bilateral swelling of the iliopsoas muscle and new-onset hematoma formation in the right psoas major muscle (Fig. 3). Hemostatic drugs were immediately administered, together with blood transfusion and supportive treatment, and the hematoma did not expand.

**Table 1:** Characteristics of laboratory examinations

Parameter	Day1	Day4	Day8	Day16	Day18	Day22	Reference value range
White cell count as × 10^9^/L	5.64	9.34	8.11	7.05	8.32	3.92	(3.5–9.5)
Platelets as × 10^9^/L	125	67	80	111	98	225	(125–350)
Hemoglobin in g/L	136	82	103	90	78	91	(130–175)
Creatine kinase in U/L	8875	8270	10,800	2448	1831	1702	(≤ 190)
ALT in U/L	125	140	119	72	56	41	(≤ 41)
AST in U/L	413	345	377	164	133	144	(≤ 41)
LDH in U/L	940	839	892	631	599	663	(135–225)
Ferritin in ug/L	2573.8	1941	1648.9	1756.8	1831	1618.2	(30–400)
myoglobin	1200	1200	1200	676	886.9	521.7	(≤ 154.9)
Urea nitrogen in mmol/L	11.37	7.7	7.1	9.1	12.9	5.7	(3.1-8.0)
Creatinine in µmol/L	73	63	60	77	73	47	(59–104)
eGFR in mL/min/1.73 m^2^	100.7	107	109.1	98.5	100.7	120.6	(> 90)
Fibrinogen in g/L	3.01	1.33	2.07	3.25	3.13	2.5	(2–4)
D-Dimer in µg/mL FEU	5.91	1.33	7.53	10.76	7.44	9.33	(< 0.5)
TT s	20.4	37.2	18	19.8	18.2	16.5	(14–19)
PT s	12.5	12.8	13.1	13.3	12.5	13.3	(11.5–14.5)
APTT s	37.4	50.2	38.5	50.1	42.6	38.8	(29–42)

In addition to the routine coagulation tests, the patient’s coagulation factors, protein C and protein S, were also tested (Additional file 2). Furthermore, special bleeding tests were carried out, including coagulation and fibrinolysis. Fibrin degradation products (FDPs) were elevated to 5.5 µg/ml, with increased thrombin-antithrombin III complex (TAT) 10.4 ng/ml (ref < 4), plasmin-α2-plasmininhibitor complex (PIC) 3.16 µg/ml (ref < 0.8) and tissue plasminogen activator-inhibitor complex (t-PAIC) 41.9 ng/ml (ref < 17). These results indicated hyperfibrinolysis, perhaps progressing to disseminated intravascular hemolytic (DIC). Fortunately, he was rescued after transfusion and hemostatic therapy with the termination of anticoagulant therapy.

**Fig. 4:**
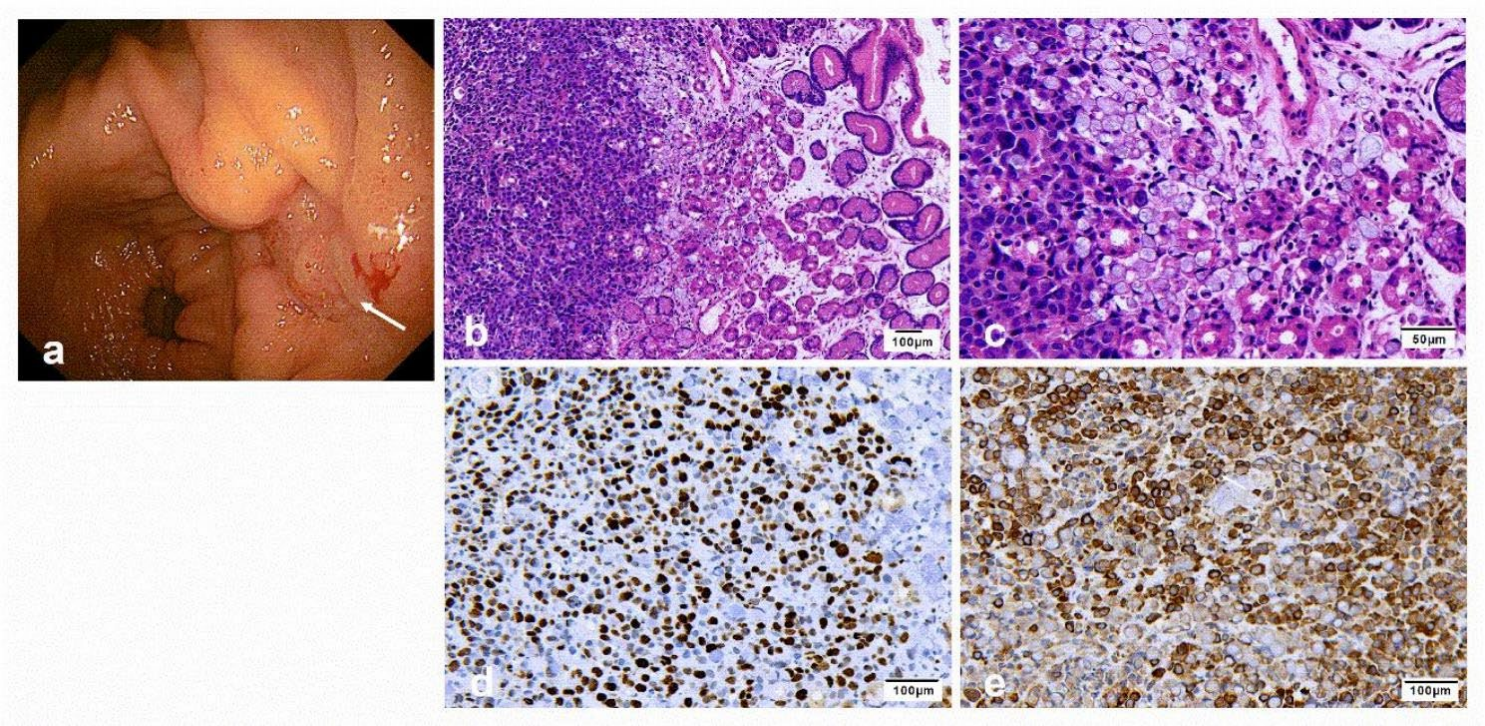
Representative images of gastric signet-ring cell carcinoma. Electronic gastroscopy showed irregular ulcer foci of approximately 2.5*2.0 cm in size with an uneven base and covered with white mucus in the posterior wall of the gastric sinus (**a**). Histological samples of the irregular ulcers in the gastric sinus (H&E 100 ×) (**b**). Histopathology of the ulcer tissue biopsy showed signet-ring cell carcinoma (white arrows) (H&E 400 ×) (**c**). Histological samples of the ulcer tissue biopsy stained for Ki67 (200 ×) (**d**), Histological samples of the ulcer tissue biopsy stained for PCK (200 ×) (**e**)

After active treatment, abdominal distension had not resolved. Electronic gastroscopy found a gastric sinus ulcer, and histopathology of the biopsy showed signet-ring cell carcinoma (Fig. 4). We rechecked CA19-9, which was > 1000 U/ml, and CEA was 24.84 ng/ml. Gastric signet-ring cell carcinoma was diagnosed, and he was transferred to the oncology department and started chemotherapy (paclitaxel 300 mg). Unfortunately, he was discharged from the oncology department and died shortly after arriving home.

## Discussion and conclusion

Spontaneous intramuscular hemorrhage (SIH) is a rare complication of DM with a high mortality rate. Orrell et al. first reported 2 cases of DM with spontaneous hemorrhage in 1998 [6]. Subsequently, a relevant literature search revealed 15 case reports of SIH in myositis between 1998 and 2022. In 2022, Xu et al. described a case series of 7 MDA5^+^ DM patients with SIH and analysed it together with previously published case reports [7]. Among cumulatively reported patients, the overall mortality was 60.9%. The poor prognostic survival was probably related to nonpalpable deep muscle involvement such as iliopsoas, and this potentially fatal complication is more likely to occur in the first 6 months after disease onset. Of the previously reported cases, two were cancer-associated DM combined with SIH. A 53-year-old female who suffered from advanced ovarian cancer involving abdominal carcinomatosis complained of weakness and myalgia in her upper and lower limbs [8]. She was diagnosed with paraneoplastic DM with elevated CK and had a positive ANA test. She received therapeutic dalteparin for cancer-associated ovarian vein thrombosis. Then, she developed multifocal hematoma affecting the left iliopsoas muscle, right flank posterior wall, paravertebral space, and thigh. Dalteparin was stopped, the patient was deemed inoperable and opted for palliative care, and she died. A 77-year-old hypertensive and diabetic male patient was diagnosed with multifocal BCLC-B stage liver cancer [9]. He experienced a recurrence of neoplasm during treatment. Heliotrope erythema, muscle weakness, and elevated CK lead to the suspicion of DM. He had received antiplatelet therapy and prophylactic LMWH, and he developed hematomas on the arms and legs. Despite active treatment, he died six months later. Our patient was diagnosed with gastric signet-ring cell carcinoma, and he also died within two months of being diagnosed with SIH. Based on these two literature reports and our case, the prognosis of cancer-associated DM complicated with SIH is extremely poor.

Patients with DM may have an increased risk of deep vein thrombosis (DVT), complicating the decision of whether to initiate anticoagulant or antiplatelet therapy [10]. According to previous reports, the use of heparin or LMWH has been considered to be a predisposing factor for DM with intramuscular hemorrhage [11–13]. In our case, the patient was treated with LMWH and pulse methylprednisolone 200 mg. After 3 days of treatment, ultrasound and MRI showed intramuscular hemorrhage from the right upper extremity. Four days after his second application of LMWH, a second hemorrhage occurred in the abdominal muscles. A CT scan showed new-onset hematoma formation in the right psoas major muscle. High-dose glucocorticoid treatment may lead to increased tissue fragility. At the same time, glucocorticoids may promote protein decomposition and inhibit protein synthesis, resulting in a negative nitrogen balance, and leading to muscle consumption. The depleted muscle tissues lose their compression and vasoconstriction effect on the peripheral blood vessels when the vessels rupture [5]. Until now, the pathophysiology of DM-associated SIH has not been elucidated. Previous case reports have speculated that SIH is associated with active inflammation of muscle-supplying vessels, high-dose glucocorticoid use, and anti-thrombotic therapy [5, 14, 15].

Malignant diseases may precede, occur concurrently with, or follow the development of the clinical signs of DM. Our patient developed DM symptoms first and was then diagnosed with gastric signet ring cell carcinoma. Futtrup et al. reported a case of metastatic gastric signet ring cell carcinoma presenting as focal myositis in the right lower extremity [16]. It is generally believed that patients with malignant tumors are in a hypercoagulable state, and are at risk of cancer-related thrombosis [17, 18]. LMWH is recommended for the management of cancer-associated thrombosis by most of the guidelines. Gastric cancer and other malignancies were reported to be associated with severe coagulation disorders in many studies. The patient’s coagulation and hemostasis systems may be strongly impacted by the presence of cancer, and the balance between clotting and bleeding is always maintained in a normal physiological state and is usually altered under disease conditions [19]. Although the risk of thrombosis is increased in most tumors, hyperfibrinolysis and hemorrhage can also be observed in some tumors. A few malignant tumors can interfere with the fibrinolytic system leading to hyperfibrinolysis and may cause bleeding [20].

Patients with dermatomyositis combined with malignancy are at dual risk of bleeding and thrombosis, and therapy with LMWH needs careful consideration. It is necessary to detect TAT, PIC and t-PAIC before anticoagulation treatment. TAT is a sensitive marker of thrombin production and an indicator of activation of the coagulation system. Its formation is the best time to judge the anticoagulant treatment, and it can be increased in the prethrombotic state. PIC is the starting point of the fibrinolysis system, which reflects the activation degree of plasmin, monitors the functional status of the fibrinolysis system, and guides the anti-fibrinolysis treatment plan. t-PAIC not only reflects abnormalities in the fibrinolysis system but is also related to endothelial damage [21]. In the present study, before the first and second episodes of hemorrhagic myositis, the level of D-dimer was significantly increased (Table 1). The continuous increase in D-dimer indicates the risk of pulmonary embolism, and anticoagulation treatment should be started. However, on the other hand, the increase in D-dimer may also indicate activation of the fibrinolytic system. In our case, TAT was elevated to 10.4 ng/ml, with increased PIC (3.16 µg/ml) and t-PAIC (41.9 ng/ml), and the TAT/PIC ratio was 2.9 × 10^− 3^. The ratio of TAT/PIC was used to estimate the fibrinolytic response to coagulation [22] and may predict vascular access failure in hemodialyzed patients after access intervention [23]. One report revealed that the TAT/PIC ratio in healthy individuals is 4.5 × 10^− 3 ^[24], and some researchers believe that this value is the fibrinolytic equilibrium point. When the TAT/PIC ratio is significantly lower than this balance point, it indicates hyperfibrinolysis, while a TAT/PIC ratio significantly higher than the balance point indicates a high risk of thrombosis. Taken together, the patient had both thrombotic risk and hyperfibrinolysis, indicating an increased risk of thrombosis and bleeding. In summary, SIH may be related to the active inflammation of blood vessels of the involved muscles in DM, the use of glucocorticoids, and hyperfibrinolysis due to tumor and anticoagulant therapy.

We reported a case of cancer-associated DM complicated by SIH. Although patients with cancer-associated DM have an increased risk of thrombosis, conventional anticoagulation therapy is not recommended. If the patient has a high risk of thrombosis and needs drug intervention, it is important to dynamically monitor the coagulation parameters during anticoagulation therapy. Especially when the D-dimer level is high, it is uncertain whether the patient is in a state of thrombosis or hyperfibrinolysis, and the detection of TAT, PIC, and t-PAIC can help to determine whether to initiate anticoagulation therapy. Further research remains to be done to elucidate the pathogenetic mechanism and the risk factors for spontaneous hemorrhagic myositis in DM.

## Electronic supplementary material

Below is the link to the electronic supplementary material.

**Additional figure S1** Swelling of the right arm The right upper limb was visibly swollen and skin bruising was visible at the elbow joint. A large number of scattered blisters were visible on the skin’s surface.

**Additional file 2** The results of coagulation factors, protein C and protein S

## Data Availability

The authors declare that data supporting the findings of this study are included in the article (and its additional files).

## Abbreviations

DM: Dermatomyositis
IIMs: Idiopathic inflammatory myopathies
SIH: Spontaneous intramuscular hemorrhage
LMWH: Low molecular weight heparin
MRI: Magnetic resonance images
CT: Computed tomography
CK: Creatine kinase
VTE: Venous thromboembolism
FDPs: Fibrin degradation products
TAT: Thrombin-antithrombin III complex
PIC: Plasmin-α2-plasmininhibitor complex
t-PAIC: Tissue plasminogen activator-inhibitor complex.
