# Supplementary material for: Spontaneous intramuscular hemorrhage in cancer-associated dermatomyositis: a case and literature review
Source: BMC Musculoskelet Disord. 2023 Jul 1;24:542. doi: 10.1186/s12891-023-06651-z (PMC10314377; doi:10.1186/s12891-023-06651-z)
Supplement: Supplementary file 1 — Additional figure S1 Swelling of the right arm The right upper limb was visibly swollen and skin bruising was visible at the elbow joint. A large number of scattered blisters were visible on the skin’s surface. [file 12891_2023_6651_MOESM1_ESM.pdf]

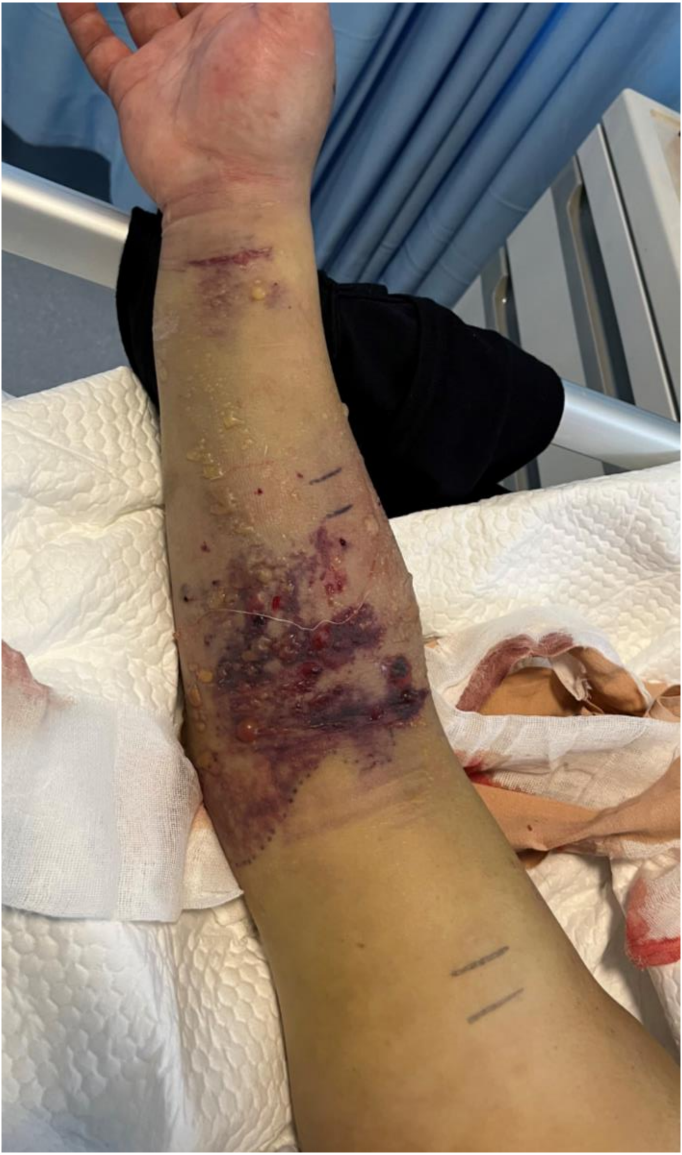

Additional figure S1 Swelling of the right arm
The right upper limb was visibly swollen and skin bruising was visible at the elbow joint. A large number of scattered blisters were visible on the skin's surface.
