# Supplementary material for: Spontaneous intramuscular hemorrhage in cancer-associated dermatomyositis: a case and literature review
Source: BMC Musculoskelet Disord. 2023 Jul 1;24:542. doi: 10.1186/s12891-023-06651-z (PMC10314377; doi:10.1186/s12891-023-06651-z)
Supplement: Supplementary file 2 — Additional file 2 The results of coagulation factors, protein C and protein S [file 12891_2023_6651_MOESM2_ESM.docx]

**The results of coagulation factors, protein C and protein S**

| **Parameter** | **Test results** | **Reference value range** |
| --- | --- | --- |
| F Ⅱ | 74.0 | (70.0-120.0) |
| F Ⅴ | 132.0 | (70.0-120.0) |
| F Ⅶ | 130.0 | (55.0-170.0) |
| F Ⅷ | 176.0 | (60.0-150) |
| F Ⅸ | 106.0 | (60.0-150) |
| F Ⅹ | 80.0 | (70.0-120.0) |
| F Ⅺ | 94.0 | (60.0-150) |
| F Ⅻ | 67.0 | (50.0-150) |
| F ⅩⅢ antigen | 73.2 | (75.2-154.8) |
| Antithrombin | 119 | (80-120) |
| VWF :RCo | 143.8 | 5--200 |
| VWF antigen | 73 | (59-104) |
| Protein C | 110 | (70-142) |
| Protein S | 63 | (77-143) |
